# Tai Chi, Yoga, and Qigong as Mind-Body Exercises

**DOI:** 10.1155/2017/8763915

**Published:** 2017-01-05

**Authors:** Yong Tai Wang, Guoyuan Huang, Gloria Duke, Yi Yang

**Affiliations:** ^1^University of Texas at Tyler, Tyler, TX, USA; ^2^University of Southern Indiana, Evansville, IN, USA; ^3^Wuhan Sports University, Wuhan, Hubei, China

Mind-body interventions or exercises may improve body function and health since nervous system affects endocrine system and immune system while performing these mind-body (MB) exercises. Tai Chi, Yoga, and Qigong are considered the most popular MB exercises, ranked by the 2002–2012 National Health Interview Surveys as the top three of the 10 most common complementary health approaches in practice.

Tai Chi is a healing/martial art combining martial art movement with Qi—vital energy circulation, breathing, and stretching techniques. Tai Chi exercise consists of a series of graceful movements with deep and slow diaphragmatic breathings performed while standing. Tai Chi exercise has been shown to have both physical and psychosocial benefits for the different populations. Yoga, a mind-body exercise, involves a combination of muscular activity and an internally directed mindful focus on awareness of the self, the breath, and energy. Yoga integrates an individual's physical, mental, and spiritual components to improve physical and mental health, particularly stress related illnesses. Qigong exercise, similar to Tai Chi, consists of a series of breath practices with body movement and meditation to attain deep focus and relaxed state. Simply speaking, Qigong exercise is practiced/used to cultivate the balance and harmony of vital energy in the human body. Considerable scientific evidence supports the health benefits of practicing Tai Chi and Qigong in various populations with differing characteristics such as age, gender, and occupation in NIH Research Report.

In this special issue, we have focused on Tai Chi, Yoga, and Qigong as mind-body exercises. Nine research articles including human experimental studies, clinical trials, and meta-analyses have been carefully reviewed, revised, and published. These research articles explore the efficacy and effectiveness of these mind-body exercises in improving, enhancing, or strengthening integrative health and well-being in relation to functional outcomes or clinical benefits on the human body. We hope you enjoy reading these research articles on this special issue.

*Yong Tai Wang* *Guoyuan Huang* *Gloria Duke* *Yi Yang*

